# Temporal-Resolution Dynamics of Polyphenolic During the Pepper Graft Healing

**DOI:** 10.3390/plants14172656

**Published:** 2025-08-27

**Authors:** Feng Zhang, Yundan Duan, Qingmao Shang

**Affiliations:** State Key Laboratory of Vegetable Biobreeding, Institute of Vegetables and Flowers, Chinese Academy of Agricultural Sciences, Beijing 100081, China; zhangfeng01@caas.cn (F.Z.); duanyundan2020@163.com (Y.D.)

**Keywords:** graft, pepper (*Capsicum annuum* L.), polyphenolic, metabolomics, accumulation profile

## Abstract

Grafting is a common agricultural technique widely used in horticulture plants to improve stress tolerance, increase yield and improve quality. However, the biochemical processes underlying graft healing, especially the temporal dynamics and specific roles of polyphenolic metabolites, remain largely unknown. Polyphenols, widely present in plants, possess many biological activities, including antioxidant, antibacterial and anti-inflammatory properties. Temporal-resolution metabolomics, a powerful tool for studying metabolite dynamics over time, offers insights into the metabolic changes during this critical phase. To better understand this process, internodes above and below the graft site were harvested from 12 time points after grafting in both non-grafted and grafted peppers for polyphenolic metabolism analyses. In this study, we describe the accumulation pattern of 432 polyphenol metabolites. By comparing with the control group, nine polyphenolics were identified that responded to graft healing. The high temporal-resolution metabolomics reported here provides a basic resource for future functional study to unravel the genetic control of pepper graft development.

## 1. Introduction

Currently, losses in horticultural production are increasing due to soil borne disease [1]. Grafting is an effective measure to overcome continuous cropping obstacles and prevent soil borne diseases, which can effectively improve horticultural plant disease resistance [2]. Grafting is used in a number of horticultural plants such as tomato [3], pepper [4], eggplant [5], cucumber [6,7], watermelon [8,9], melon [10], grape [11], apple [12] and citrus [13]. Graft healing is complex as it involves a series of cytological events including callus formation, cell division, cell differentiation, cell wall synthesis, vascular bundle regeneration and reconnection [14,15,16,17,18,19,20]. However, despite many studies of plant grafting, the mechanisms underlying the cohabitation of the scion and rootstock are still poorly understood.

Pepper belongs to the Solanaceae family and is an annual herbaceous plant widely planted around the world. According to FAO statistics, the planting area of chili peppers worldwide in 2018 was 1.9904 million hectares, the annual sowing area of chili peppers in China accounts for about the world’s annual sowing area of chili peppers at 39% (https://www.fao.org/faostat/). Chili cultivation has high economic benefits and commercial value, but Chili peppers are susceptible to many diseases and pests during the production process. Especially with the increasing area of pepper facility cultivation year by year, soil-borne diseases such as pepper blight and root rot caused by continuous cropping are becoming more and more severe. Grafting is widely used to increase pepper resistance and improve plant growth vigor to increase productivity in stress environments such as salt, alkalinity, drought, heat and chilling [21]. So far, most research into the mechanism of graft healing has been mainly focused on the model plants like rice, tomato, and Arabidopsis. The mechanisms of grafting in pepper are still poorly understood.

The metabolites play important roles in plant growth, development and adaptation to environmental changes [22,23]. Metabolomics is a powerful technology for identifying small-molecule metabolites and an effective methodology for analyzing the dynamics of metabolites exposed to endogenous or exogenous factors in plants [24]. Specifically, gas chromatography–mass spectrometry and liquid chromatography–mass spectrometry have been used to analyze the chemical composition of plants [25]. In tomato, more than 900 metabolites were identified in the pericarp tissue of ripe fruits [26]. In another report, 540 metabolites were identified in different tissues; the accumulation of the 540 metabolites during tomato development is tissue specific [27]. In pepper, 1100 metabolites were detected including 456 flavonoids, 422 acyclic diterpenoids, 153 other phenolics and 59 other compounds, including lipids and fatty acids [28]. In another report, transcriptomic, metabolomic and hormonal analyses were used to investigate the molecular mechanism of yellowing in the yl1 pepper mutant, a total of 28 carotenoids were detected in both lines upon exposure to high, medium and low light [29]. However, to the best of our knowledge, no comprehensive investigation has been conducted regarding the metabolic response in pepper during graft healing.

Polyphenolic compounds are widely present in plants such as fruits, vegetables, grains and beans. Phenolic acid structures with phenolic and organic acid functional groups are mostly C6-C1 skeleton hydroxybenzoic acid derivatives and C6-C3 skeleton hydroxycinnamic acid derivatives. Common hydroxybenzoic acid types include gallic acid, tannic acid and ginkgoid acid. Hydroxycinnamic acid types include chlorogenic acid, caffeic acid and salvianolic acid. Phenolic compounds of various molecules including phenolic acids, flavonoids, lignans, coumarins and tannins are important secondary metabolites in plants [30]. Phenolic acid compounds have diverse effects and possess biological activities such as antibacterial, antiviral and antioxidant properties [31]. In grafting, polyphenolic compounds are known to be biochemical markers of incompatible grafts without vascular bundle reconnection [32]. The polyphenolic compounds are deposited in the cell walls of compatible combinations, but are transported into vacuoles in incompatible combinations [33]. However, despite many studies of polyphenols, the temporal dynamics and specific roles of polyphenolic compounds during pepper graft healing remain unexplored.

To better understand the accumulation mode and role of polyphenolic compounds during the pepper graft healing, we constructed a high-temporal-resolution metabolome dataset from graft union of grafted pepper at different healing stages. Internodes from the main stem above and below the graft site were harvested from 12 time points after grafting in both non-grafted and grafted peppers for polyphenolic metabolism analyses. We detected 432 annotated polyphenolic metabolites in 174 different tissues and stages. A total of 53 differentially accumulated polyphenolics metabolites during pepper graft healing were ultimately obtained, including 34 phenolic acids, 14 flavonoids, 2 lignans, 2 coumarins and 1 tannins. Finally, nine polyphenolics were identified that responded to graft gealing. Collectively, our findings provide a basis to explain polyphenolic accumulation during pepper graft healing.

## 2. Results

### 2.1. The Generation of Pepper Graft Healing Time-Course Polyphenolics Metabolome Data

**Figure 1:**
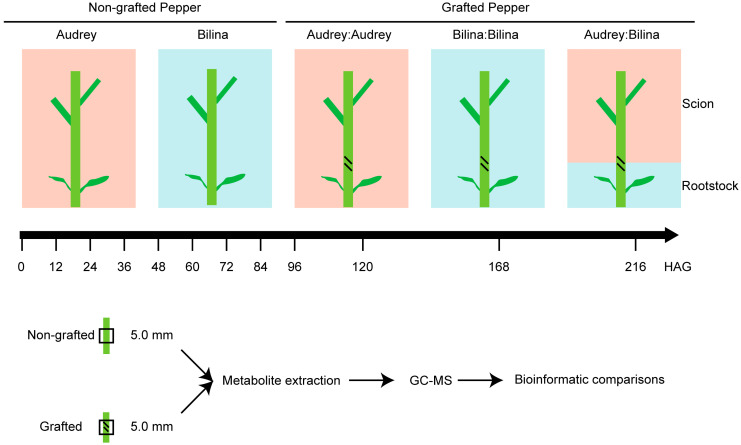
Schematic representation of the design for the metabolome data.

**Figure 2:**
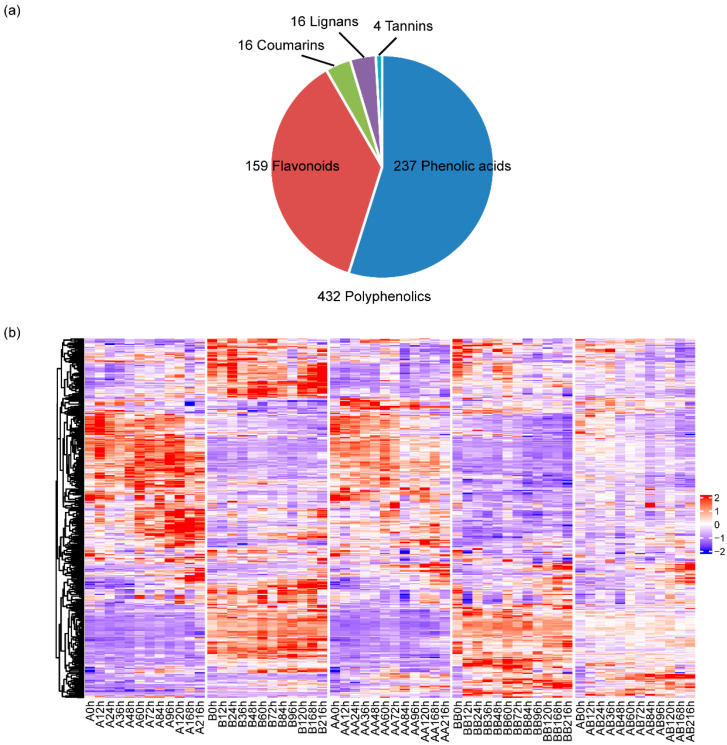
Summary of metabolome data. (**a**) A distribution of different types of 432 polyphenolic. (**b**) Hierarchically clustered heatmap of the 432 annotated metabolites from 174 samples.

To understand the process behind pepper graft healing and to identify the differential accumulation of polyphenolics, we generated the polyphenolics metabolome dataset (Figure 1). The grafting survival rate was 100% in both grafted groups. For metabolome analysis, samples were analyzed using a broadly targeted LC–tandem MS (LC-MS/MS)-based metabolic profiling method. A total of 432 distinct annotated polyphenolics metabolites were identified in at least one tissue, including 237 phenolic acids, 159 flavonoids, 16 lignans, 16 coumarins and 4 tannins (Figure 2a). The relative content of these 432 metabolites was detected between grafted and non-grafted samples at each time point (Figure 2b). Through the analysis of the content of polyphenolic metabolites, we found that there were significant differences in the content of polyphenolic metabolites among different varieties.

Schematic representation of the design for the graft healing in *Capsicum annuum*. Fifty-eight samples were collected for metabolic profiling. The axis indicates the sample harvest data. HAG: hours after graft. Audrey: a scion variety. Bilina: a rootstock variety.

**Figure 3:**
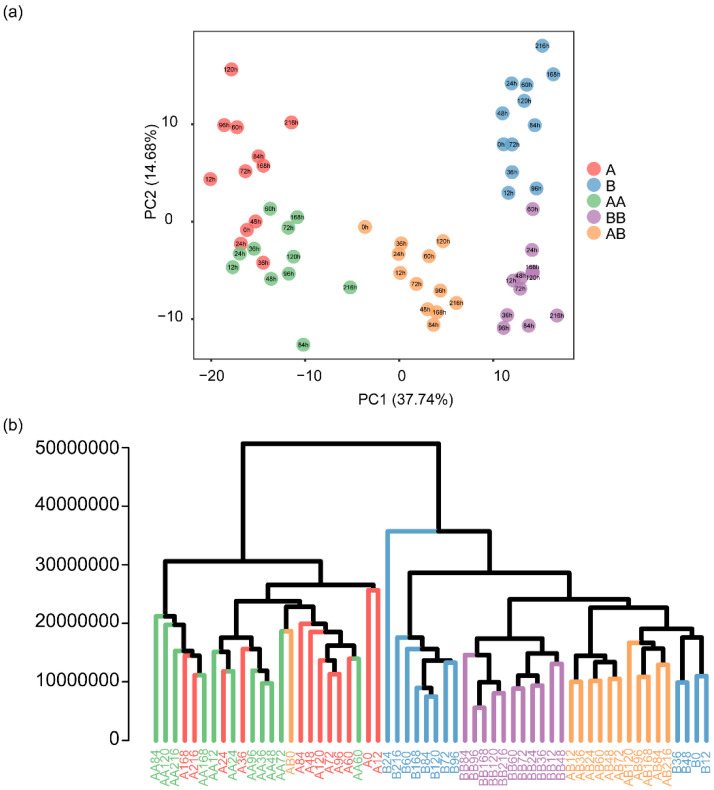
Cluster analysis of metabolome data. (**a**) PCA of metabolome data from the 174 samples. (**b**) Cluster dendrogram of metabolome data from the 174 samples. Red represents Group A, light green represents Group AA, blue represents Group B, purple represents Group BB, and orange represents Group AB. The number in the circle represent time points.

To obtain profiles of differential metabolites, we performed a PCA of the identified metabolites across the twelve time points and established that 432 metabolites could be further divided into five groups (Figure 3a). Notably, metabolites in ‘Audrey’ and ‘Bilina’ were clearly separated from each other (corresponding to PC1), demonstrating the existence of major metabolomic differences between them. The metabolomes between the two autologous grafting were more different than between the allogeneic grafting, which were in the middle. The separation of metabolites between non-grafted groups and grafted groups corresponds to PC2. In line with the PCA, the cluster dendrogram could also be divided into five independent subgroups (Figure 3b). These results suggest that polyphenolic metabolite accumulation shows significant specificity during pepper graft healing.

### 2.2. Accumulation Profiles of the Metabolites Involved in Phenylpropanoid Biosynthesis

**Figure 4:**
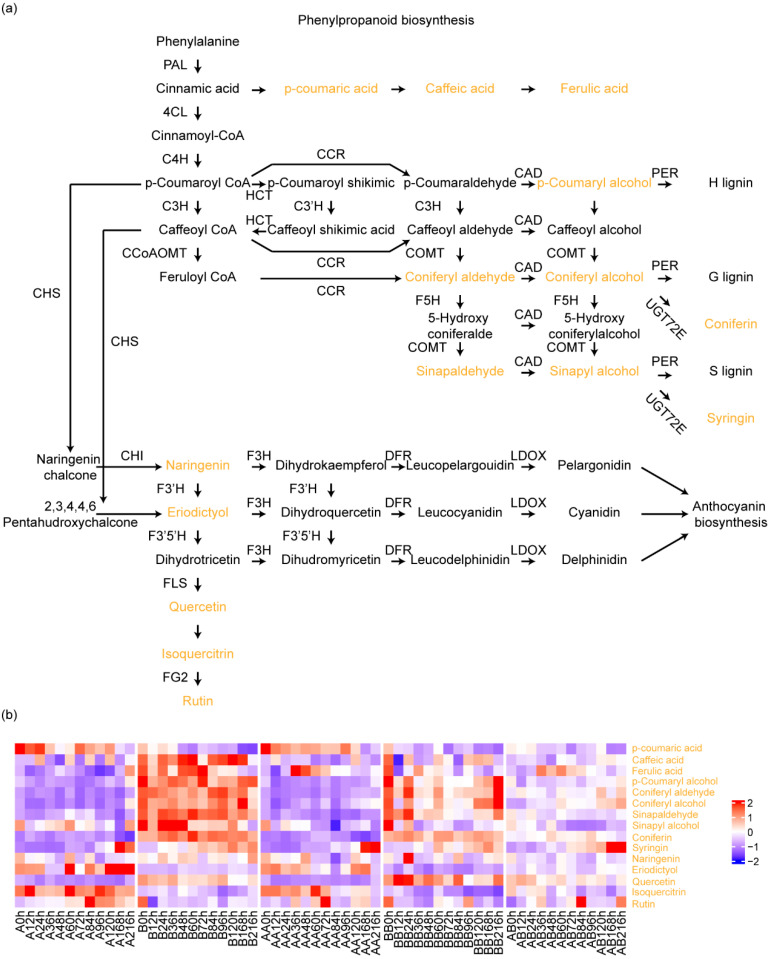
Accumulation profiles of the metabolites involved in phenylpropanoid biosynthesis. (**a**) Schematic representation of phenylpropanoid biosynthesis pathway. The metabolites detected in this study are marked with colors. (**b**) Heatmap of the 15 detected metabolites involved in phenylpropanoid biosynthesis from 174 samples.

To examine the high temporal-resolution metabolomics report here which provides an important resource for future study to unravel the control of graft development, we first tested it with a previously known regulatory network phenylpropanoid biosynthesis pathway. This pathway originates with the amino acid phenylalanine. Downstream of the gateway enzymes, phenylalanine ammonia-lyse and cinnamic acid 4-hydroxylase, the lignin/flavonoid pathway consists of a series of enzymes that catalyze diverse reactions, leading to the biosynthesis (Figure 4a). Through the analysis of the 15 detected metabolite content in the phenylpropane synthesis pathway (Figure 4b), we found that there were significant differences in the content of phenolic acid metabolites among different varieties, and different developmental stages also significantly affected the content of phenolic acid metabolites among varieties. These results suggest that grafting does not affect the main pathway of phenylpropane metabolism.

### 2.3. Differential Metabolite Screening During Pepper Graft Healing

**Figure 5:**
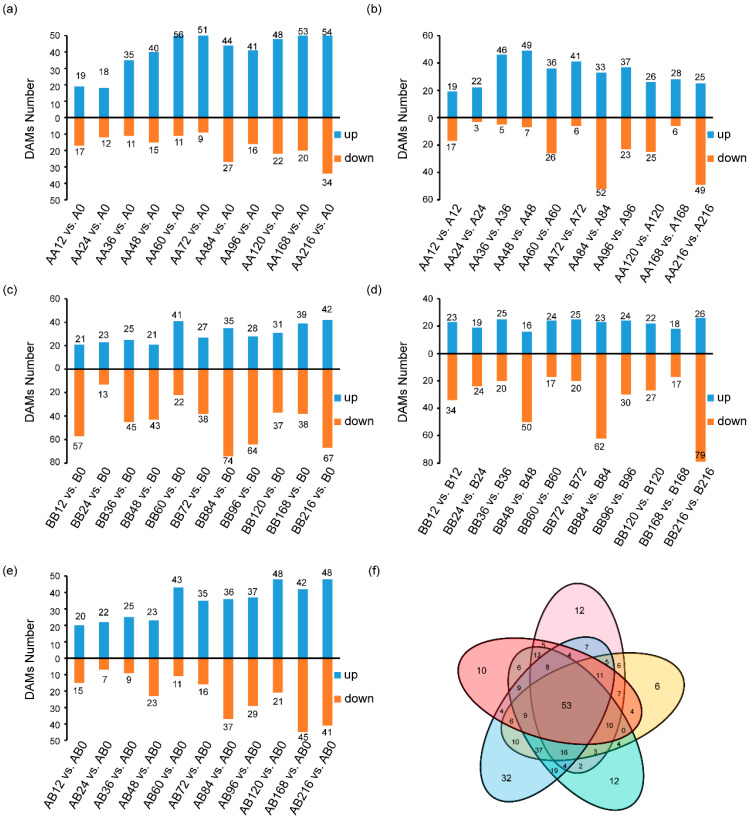
Screening of differentially accumulated metabolites (DAMs). (**a**–**e**) Number of differential metabolites in different comparison groups. (**f**) Venn plot of 5 groups of differentials accumulated metabolic numbers.

**Figure 6:**
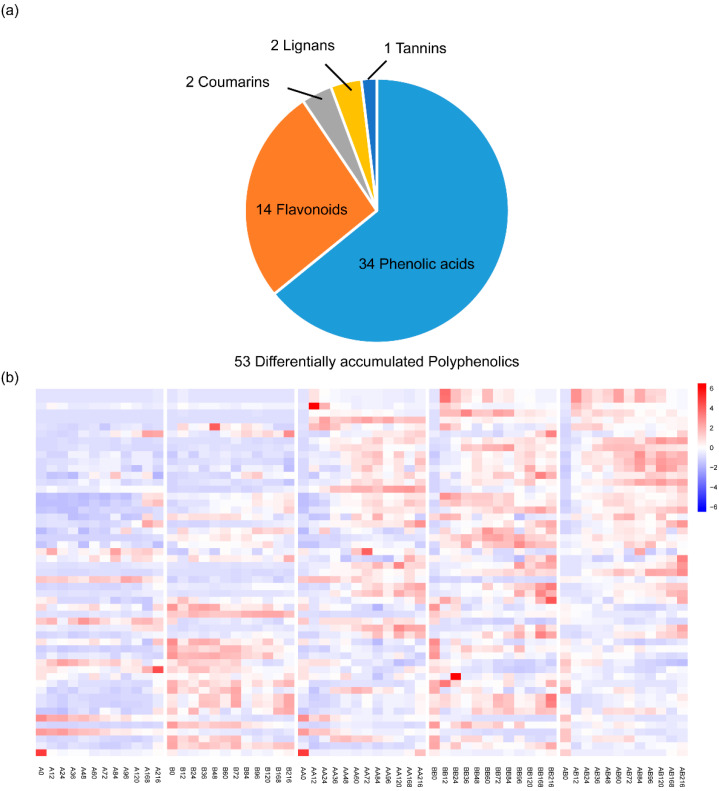
A distribution of differentially accumulated 53 polyphenolics. (**a**) A distribution of different types of 53 polyphenolic. (**b**) Hierarchically clustered heatmap of the 53 annotated metabolites from 174 samples.

In order to more accurately screen the key polyphenolic metabolites involved in the healing process of scion and rootstock allografts, we set up strict controls, including a non-graft control group and autograft control group, hoping to eliminate the effects of different genetic backgrounds and developmental stages of scions and rootstocks. We compared the differential accumulated metabolites between different time points and 0 h after grafting (Figure 5a,c,e), and also compared the differential accumulated metabolites between the grafting group and the non-grafting group (Figure 5b,d). By taking the intersection, 53 differentially accumulated polyphenolic metabolites were ultimately obtained (Figure 5f), including 34 phenolic acids, 14 flavonoids, 2 lignans, 2 coumarins and 1 tannins (Figure 6).

### 2.4. The Responded Polyphenolics During Pepper Graft Healing

**Figure 7:**
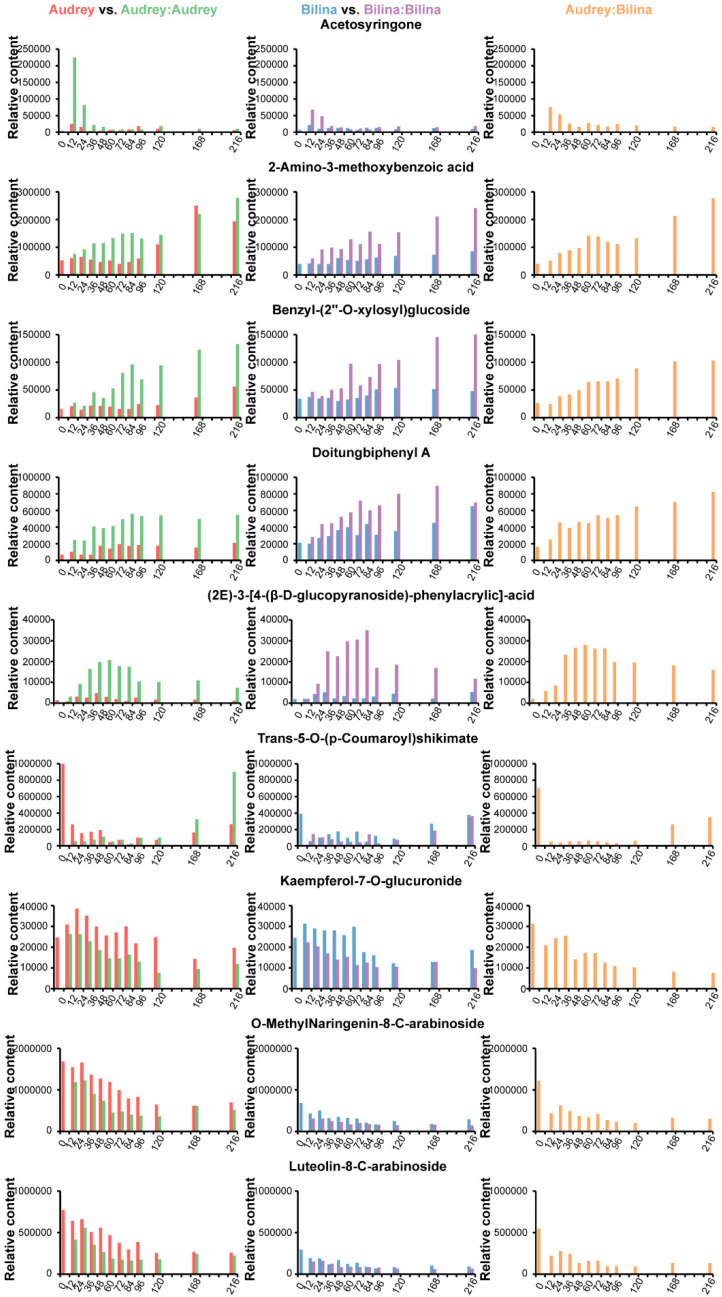
Nine responded polyphenolics during pepper graft healing: red represents Group A, light green represents Group AA, blue represents Group B, purple represents Group BB, and orange represents Group AB. Relative content was based on metabolomics.

The developmental progress of graft union roughly includes wound response, isolation layer formation, initial adhesion between scion and rootstock, formation of the wound callus and plasmodesmata, and vascular reconnection [34]. To find the responded polyphenolics during pepper graft healing, we checked the accumulation profiles of the 53 differentially accumulated polyphenolics one by one. Finally, we found nine polyphenolic regular changes during pepper graft healing (Figure 7). Five polyphenolics were induced during graft healing, while four polyphenolics decreased after grafting. The relative content of Acetosyringone peaked at 12 HAG in Group AA, Group BB and Group AB and immediately decreased at later time points. 2-Amino-3-methoxybenzoic acid, Benzyl-(2″-O-xylosyl) glucoside, Doitungbiphenyl A and (2E)-3-[4-(β-D-glucopyranoside)-phenylacrylic]-acid show similar accumulation patterns, induced in the middle during graft healing. Trans-5-O-(p-Cournaroyl) shikimate, Kaempferol-7-O-glucuronide, O-MethylNaringenin-8-C-arabinoside and Luteolin-8-C-arabinoside were decreased after grafting. These findings indicate that these metabolites could have reliable functions in pepper during graft healing.

## 3. Discussion

The mechanism of graft healing is mainly focused on the model plants like rice, tomato and Arabidopsis. Lots of transcriptomes have been carried out with these plants and enriched our understanding of the grafting healing process. In Arabidopsis, auxin was found to activate the vascular regeneration under graft healing [17]. Many Arabidopsis and rice orthologue genes have responded in the same way to grafting [20]. Paclobutrazol (PBZ), an inhibitor of gibberellin biosynthesis, or the auxin transport inhibitor 2,3,5-triiodobenzoic acid (TIBA) significantly reduced graft union formation, whereas application of gibberellin and auxin enhanced graft formation in rice [20]. This indicates that monocotyledonous and dicotyledonous plants have conservation in grafting healing. There is still a lot that is unknown about whether there are other metabolites involved in graft healing. Polyphenols, widely present in plants, possess various biological activities, including antioxidant, antibacterial and anti-inflammatory properties [35]. During vegetable grafting, polyphenols may play pivotal roles through multiple mechanisms; research specifically on their functions in vegetable grafting is relatively limited. The present study based on a widely target metabolomic approach provides insights into the metabolic profiles in pepper during graft healing. We detected 432 annotated polyphenolic metabolites in 174 different tissues and stages during pepper graft healing, including 237 phenolic acids, 159 flavonoids, 16 lignans, 16 coumarins and 4 tannins. Finally, we identified nine polyphenolics that responded to graft gealing. To the best of our knowledge, no comprehensive investigation has been conducted regarding the polyphenol metabolic response in pepper during graft healing.

Previous studies have focused on utilizing metabolomics techniques to investigate the developmental traits of grafted scions [36] and their impact on fruit quality [37], but research on graft healing is relatively limited. In pear, several flavonols could be involved in graft incompatibility [32]. In grape, by study metabolites across different scion/rootstock combinations, they found stilbenes can be used as a good early marker of grafting success to determine the short-term compatibility [38,39]. In our study, we found nine polyphenolics regular changes during pepper graft healing. Based on the known functions of polyphenols, we can summarize their potential effects. The antioxidant properties of polyphenols may play a crucial role in vegetable grafting. The plant tissue undergoes some degree of damage during the grafting process [15], which may lead to oxidative stress. The reactive oxygen species scavenging showed significant differential expression during graft healing [40] and oxidative detoxification enzymes accumulate to high levels near the graft region [41]. As effective antioxidants, polyphenols can scavenge or neutralize free radicals, reducing oxidative stress damage to plant cells, thereby promoting wound healing at the grafting site and facilitating plant recovery. Previously, we also found L-phenylalanine and lignin metabolism genes were expressed at higher levels in grafted tissues than in control tissues [42]. The antibacterial properties of polyphenols may help reduce disease infection during grafting. The plant tissue is exposed to the external environment during the grafting process, making it susceptible to pathogen attack. Polyphenols have broad-spectrum antibacterial activity, inhibiting the growth and reproduction of multiple pathogens [43], thereby lowering the incidence of disease after grafting and improving grafting survival rates. Furthermore, polyphenols may influence plant growth and development after grafting by regulating signal transduction and gene expression within the plant. For example, phenolic acid-induced phase separation and translation inhibition mediate plant interspecific competition [44]. Polyphenols participate in the plant’s redox signaling network, interacting with other signaling molecules to regulate plant growth and defense responses. During grafting, polyphenols may coordinate the growth and development of the rootstock and scion by affecting signal transduction and gene expression between them, thereby enhancing the adaptability and stress resistance of grafted plants.

It is important to acknowledge several limitations of this study. First and foremost, the sample size employed in this study was relatively small. This inherent constraint may severely limit the generalizability of our findings. A small sample size may not adequately represent the entire population or the full range of possible variations in the biological processes under investigation. For instance, the observed patterns of polyphenolic metabolite changes might be specific to the particular samples used and may not hold true for a broader range of vegetable species or grafting conditions. The experimental design also presents limitations. The use of only one scion–rootstock combination restricts the scope of the study. Grafting is a complex process influenced by various genetic and physiological factors of both the scion and the rootstock. By relying on a single combination, the study lacks broader comparisons. To truly demonstrate the universality of candidate metabolites and change patterns, more diverse grafting combinations should be selected in future research. This would allow for a more comprehensive understanding of how polyphenolic metabolites behave across different genetic backgrounds. Secondly, the focus of our study was solely on the mixed grafting joint site condition. While this provides valuable initial insights, future studies that examine the distinct content of metabolites in scions and rootstocks separately would be of great value. Understanding the individual contributions and changes in metabolite levels in each part could shed more light on the overall grafting process and the role of polyphenols. Despite these limitations, our findings are not without merit. They provide important insights into understanding the temporal changes of polyphenolic metabolites during the grafting healing process. This knowledge is a step forward in unraveling the complex biochemical events that occur during grafting.

Although polyphenols are known to have multiple potential roles in vegetable grafting, such as antioxidant activity, defense against pathogens and involvement in cell–cell communication, the specific mechanisms underlying their functions during grafting are not fully understood. Future research can delve deeper into the mechanisms of action of polyphenols in vegetable grafting. For example, how do they interact with other signaling molecules or enzymes during the healing process? Moreover, future research can explore how to optimize grafting techniques by regulating the synthesis and metabolism of polyphenols. By manipulating factors that influence polyphenol production, such as environmental conditions or genetic modifications, we may be able to improve grafting survival rates and plant growth performance. For instance, if we can enhance the synthesis of beneficial polyphenols at the grafting site, it might promote better tissue integration and reduce the risk of graft failure. In summary, as compounds with multiple biological activities, polyphenols may play important roles during vegetable grafting. By delving into the mechanisms of action of polyphenols during grafting, we can provide new insights and methods for optimizing grafting techniques, improving grafting survival rates and enhancing plant growth performance. Overcoming the limitations of the current study through future research will undoubtedly lead to a more comprehensive understanding of vegetable grafting and the role of polyphenols in this vital agricultural practice.

## 4. Materials and Methods

### 4.1. Plant Material

The scion variety ‘Audrey’ (from Syngenta, Shouguang, China) and the rootstock variety ‘Bilina’ (from Qingdao Goldenmama, Qingdao, China) were used in this study. The rootstock is sown 5–10 days earlier than the scion, with 5–7 true leaves on the rootstock and 5–6 true leaves on the scion. Grafting is carried out when the stem thickness is 2–4 mm. We performed grafting and cut the internodes from the main stem between the first true leaves and the cotyledon at an angle of 45°, then reattached the two parts and fixed with graft tubes. Within 3 days after grafting, seal and avoid light. The temperature should be controlled at 25–27 °C during the day and 18–21 °C at night, with an air humidity of over 90%. After 3 days, increase the light appropriately, and start ventilation after 5 days. After 1 week, the film can be completely peeled off.

We established five groups for comparison: a non-grafted control for scion variety ‘Audrey’ (Group A), a non-grafted control for rootstock variety ‘Bilina’ (Group B), a self-grafted control for scion variety ‘Audrey’ (Group AA), a self-grafted control for rootstock variety ‘Bilina’ (Group BB) and an experimental group featuring heterologous grafting between scion ‘Audrey’ and rootstock ‘Bilina’ (Group AB). The grafting survival rate was 100% in both grafted groups. Samples were collected from all major graft healing stages of pepper at 0, 12, 24, 36, 48, 60, 72, 84, 96, 120, 168 and 216 h after grafting (HAG), with a total of 12 time points. We collected 5 mm tissues both above and below the graft junction, and we collected 10 mm internodes from the non-grafted control. All the samples were obtained from the main stem, and the wound area was not avoided. There were three biological replicates, each of which was a pooled sample from ten plants. Three replicates from three different acupoints were randomly sampled. We collected 174 samples from five groups, including both non-grafted and grafted peppers.

### 4.2. Sample Preparation and Extraction Process

Biological samples undergo freeze-drying using a vacuum freeze-dryer (Scientz-100F, Ningbo, China). Subsequently, the freeze-dried sample is ground into a fine powder using a mixer mill (MM 400, Retsch, Hanau, Germany) equipped with a zirconia bead, operating at 30 Hz for 1.5 min. To prepare the extract, 100 mg of the lyophilized powder is dissolved in 1.2 mL of a 70% methanol solution. The mixture is then vortexed for 30 s every 30 min, repeated six times in total, and left to stand in a refrigerator at 4 °C overnight. After centrifugation at 12,000 rpm for 10 min, the supernatant is filtered through a 0.22 μm pore size filter (SCAA-104; ANPEL, Shanghai, China, http://www.anpel.com.cn/) prior to UPLC-MS/MS analysis.

### 4.3. UPLC Analytical Conditions

The sample extracts are subjected to analysis using a UPLC-ESI-MS/MS system, which consists of a UPLC unit (SHIMADZU Nexera X2, Kyoto, Japan) and a mass spectrometer (Applied Biosystems 4500 Q TRAP, Carlsbad, CA, USA). The UPLC analysis is conducted under the following conditions: the column used is an Agilent SB-C18 (1.8 µm, 2.1 mm * 100 mm). The mobile phase comprises solvent A, which is pure water containing 0.1% formic acid, and solvent B, which is acetonitrile with 0.1% formic acid. Sample measurements are carried out using a gradient elution program starting with 95% A and 5% B. Over the course of 9 min, a linear gradient is programmed to reach 5% A and 95% B, which is maintained for 1 min. Subsequently, the composition is adjusted back to 95% A and 5.0% B within 1.1 min and held constant for an additional 2.9 min. The flow rate is set at 0.35 mL per minute, the column oven temperature is maintained at 40 °C, and the injection volume is 4 μL. The effluent from the UPLC is then connected to an ESI-triple quadrupole-linear ion trap (QTRAP)-MS for further analysis.

### 4.4. ESI-Q TRAP-MS/MS Analysis

Both linear ion trap (LIT) and triple quadrupole (QQQ) scans are performed on a triple quadrupole-linear ion trap mass spectrometer (Q TRAP), specifically the AB4500 Q TRAP UPLC/MS/MS System. This system is equipped with an ESI Turbo Ion-Spray interface and can operate in both positive and negative ion modes, controlled by Analyst 1.6.3 software (AB Sciex). The operational parameters for the ESI source are as follows: the ion source is set to turbo spray mode with a source temperature of 550 °C. The ion spray voltage (IS) is set at 5500 V for positive ion mode and −4500 V for negative ion mode. The ion source gases I (GSI) and II (GSII), as well as the curtain gas (CUR), are set at 50, 60 and 25.0 psi, respectively. The collision-activated dissociation (CAD) is set to a high level. Instrument tuning and mass calibration are performed using 10 and 100 μmol/L polypropylene glycol solutions in QQQ and LIT modes, respectively. QQQ scans are conducted as MRM experiments with the collision gas (nitrogen) set to medium. The declustering potential (DP) and collision energy (CE) for individual MRM transitions are further optimized. A specific set of MRM transitions is monitored for each period based on the metabolites eluted during that time frame.

### 4.5. Statistical Analysis

To ensure the reliability and accuracy of the experimental data, all assays were carried out in triplicate. The results were then processed and presented as the median value along with the standard deviation (SD), utilizing Microsoft Excel (Microsoft 365, Microsoft Corporation, Redmond, WA, USA) for calculations. For in-depth data exploration and pattern recognition, principal component analysis (PCA) and hierarchical clustering analysis (HCA) were performed. These analyses were conducted using the R software 4.2.1, which is freely available at https://www.r-project.org/. PCA helps in reducing the dimensionality of the data while retaining its essential features, enabling us to visualize the overall structure and relationships among samples. HCA, on the other hand, groups similar samples or variables together based on their characteristics, providing insights into the hierarchical organization of the data. To determine the statistical significance of differences between groups, a significance level of *p* < 0.05 was set. The analysis was performed using one-way analysis of variance (ANOVA) in the commercial software SPSS 24.0. One-way ANOVA is a robust statistical method for comparing the means of three or more groups, allowing us to assess whether there are any statistically significant differences among them.

## 5. Conclusions

As compounds with multiple biological activities, polyphenols may play important roles during vegetable grafting. The present study based on a widely target metabolomic approach provides insights into the metabolic profiles in pepper during graft healing. We detected 432 annotated polyphenolic metabolites in 174 different tissues and stages during pepper graft healing, including 237 phenolic acids, 159 flavonoids, 16 lignans, 16 coumarins and 4 tannins. Fifty-three polyphenolic metabolites were identified as differential accumulated metabolites by comparing the non-grafted group, the autograft group and the allograft group. In addition, nine polyphenolic metabolites, including acetosyringone, 2-Amino-3-methoxybenzoic acid, Benzyl-(2″-O-xylosyl) glucoside, Doitungbiphenyl A, (2E)-3-[4-(β-D-glucopyranoside)-phenylacrylic]-acid, Trans-5-O-(p-Cournaroyl) shikimate, Kaempferol-7-O-glucuronide, O-MethylNaringenin-8-C-arabinoside and Luteolin-8-C-arabinoside, were identified as key metabolites. The key metabolites may play roles in processes such as cell wall reconstruction and signal transduction. It has application potential in pepper graft healing. By delving into the mechanisms of action of polyphenols during grafting, we can provide new insights and methods for optimizing grafting techniques, improving grafting survival rates and enhancing plant growth performance.

## Data Availability

All data in this study can be found in the manuscript.
